# Multiscale Transfer of Cohesive-Zone Parameters for Opening-Dominated Interlaminar Fracture in Carbon-Fiber-Reinforced Aluminum Laminates

**DOI:** 10.3390/polym18182287

**Published:** 2026-09-19

**Authors:** Jiangwen Chen, Chaoqun Liang, Xin Luo

**Affiliations:** 1 School of Advanced Manufacturing, Nanchang University, Nanchang 330031, China; 2 Department of Metal Forming Technologies, Bauman Moscow State Technical University, 105005 Moscow, Russia

**Keywords:** carbon-fiber-reinforced aluminum laminates, opening-dominated interlaminar fracture, cohesive zone model, multiscale modeling, molecular dynamics

## Abstract

Specimen-scale fitting can reproduce interlaminar fracture in carbon-fiber-reinforced aluminum laminates (CARALL) but obscures the physical origin of cohesive-zone parameters. We present a multiscale framework integrating molecular dynamics (MD), a representative volume element (RVE), finite element (FE) modeling, and opening-dominated asymmetric double-cantilever-beam (DCB) tests. At modeled high rates, an ideal nonbonded Al/epoxy interface exhibited normal and tangential strengths of 470.09 and 352.93 MPa, respectively. Across 0.001–0.005 Å/fs, normal and tangential peak tractions increased by 5.93% and 5.82%, respectively, whereas traction-separation integrals varied nonmonotonically. These single-atomistic-realization descriptors were transferred to an RVE containing Al/matrix and fiber/matrix interfaces. In this morphology, fiber/matrix debonding preceded Al/matrix damage in all three realizations, and the RVE yielded mean effective normal and tangential strengths of 27.42 ± 0.33 and 39.04 ± 0.65 MPa, together with mean Mode I and Mode II fracture energies of 0.36 ± 0.02 and 0.81 ± 0.04 N/mm, respectively, where the means and standard deviations are taken over the three stochastic fiber realizations. The RVE-derived strengths and fracture energies were assigned directly to the DCB model without fitting the experimental response. The FE peak load was 44.48 N, 5.50% above the four-specimen mean of 42.16 ± 1.22 N, and the predicted damage location was qualitatively consistent with the observed Al/matrix interfacial damage. Because the interface model is idealized and the comparison rests on load–displacement data without synchronized crack-length measurements or independent fracture-resistance data, these results are reported as a configuration-specific assessment of the transfer procedure rather than as a quantitative validation; the transferred parameters are not intended to predict the chemically and structurally complex anodized interface.

## 1. Introduction

Fiber–metal laminates combine the ductility and damage tolerance of metals with the high specific stiffness and strength of fiber-reinforced composites [1,2,3]. These attributes make them attractive for lightweight load-bearing structures in transport, aerospace and protective engineering. Carbon-fiber-reinforced aluminum laminates (CARALL) can provide greater stiffness and load-carrying efficiency than glass- or aramid-fiber systems [4,5]. Their reliability, however, is governed by the interlaminar region. Mechanical mismatch between aluminum and carbon-fiber-reinforced polymer (CFRP) concentrates stress and promotes interfacial debonding and progressive delamination [6,7]. Experimental studies have shown that aluminum surface treatments, carbon-nanotube-modified interfaces, and cellulose interlayers can alter the interlaminar fracture resistance of CARALL and related fiber-metal laminates [8,9,10,11,12]. Recent work has also characterized Mode I and Mode II interlaminar strength and fracture toughness in co-cured fiber-metal laminates [13]. Predicting interlaminar fracture is therefore central to the design and damage assessment of CARALL structures.

Molecular dynamics (MD) simulations can probe metal/polymer interfaces that are difficult to characterize experimentally [14,15,16,17]. Adhesion at polymer/aluminum interfaces depends on adsorption geometry, chain mobility, nonbonded interactions, oxide structure, and surface functional groups [18,19,20,21,22]. Thermal-oxidative aging and interfacial water can further alter adhesion and fracture [23,24,25]. Recent combined atomistic and experimental evidence shows that inorganic surface chemistry can modify epoxy structure and fracture behavior [26]. Traction–separation relations derived from MD are increasingly used to estimate interfacial stiffness, strength, and fracture energy for continuum models [27,28,29,30,31,32,33,34,35]. However, an ideal, nonbonded atomistic interface cannot directly represent the chemistry, morphology, or quasi-static behavior of an anodized experimental interface.

At the structural scale, cohesive-zone models (CZMs) represent damage initiation, progressive softening, and complete decohesion within a single constitutive framework [36,37,38]. Recent formulations address Mode I and mixed-mode delamination through multilinear, mechanism-based, temperature-dependent, and rate-dependent laws [39,40,41,42,43,44]. They have also been extended to fatigue growth, interacting cracks, and progressive failure in composite laminates and hybrid joints [45,46,47,48,49]. Direct and inverse experiments have identified cohesive laws under Mode I, Mode II, and mixed-mode loading, but the resulting parameters remain sensitive to measurement uncertainty, specimen kinematics, and the adopted identification procedure [50,51,52,53,54,55,56]. Moreover, structural cohesive parameters are generally inferred from coupon- or specimen-scale responses, which obscures their lower-scale physical origin and limits transfer between material systems and structural configurations.

Multiscale modeling could connect intrinsic interface physics to structural fracture predictions [57,58]. Atomistically informed cohesive laws have been developed for carbon-fiber, carbon-nanotube, graphene/epoxy, polymer/alumina, and other heterogeneous interfaces [32,33,34,35,59,60,61,62,63]. Multiscale studies of polymer-matrix composites further show that heterogeneity, constraint, surface chemistry, and interface competition can modify lower-scale properties [26,63,64,65,66,67,68]. Such mesoscale conversion may be particularly important in CARALL. The Al/matrix interface carries the primary through-thickness load, whereas the fiber/matrix interface redistributes local stress and develops damage within the composite. A single ideal interface is therefore unlikely to represent the effective interlaminar response adequately.

Existing studies have characterized interlaminar fracture and interface modification in CARALL, identified cohesive laws from specimen-level tests, or transferred atomistic responses to continuum interface models. However, a CARALL-specific study combining an ideal Al/epoxy atomistic interface, a heterogeneous two-interface RVE, and a structural DCB comparison remains limited. Here, bottom-up transfer denotes the sequential use of idealized atomistic descriptors, RVE-based conversion, and structural assignment without fitting the DCB response. It does not imply that the atomistic model directly reproduces the chemistry, morphology, or quasi-static behavior of the anodized experimental interface. We therefore examine this route as a configuration-bounded assessment of the transfer procedure, in which the structural comparison serves as a descriptive check on the transferred values rather than as a validation of the workflow. MD supplies model-specific Al/epoxy descriptors under normal and tangential loading, and a three-phase RVE containing three stochastic fiber realizations converts them into effective cohesive strengths and fracture energies. Four opening-dominated asymmetric DCB tests and a corresponding FE model then provide a configuration-specific comparison without specimen-level inverse fitting, in which the predicted load level and damage location are compared descriptively under the idealizations of the atomistic interface and the absence of synchronized crack-length and independent fracture-resistance data.

## 2. Methods

### 2.1. Multiscale Framework and Parameter-Transfer Strategy

**Figure 1:**
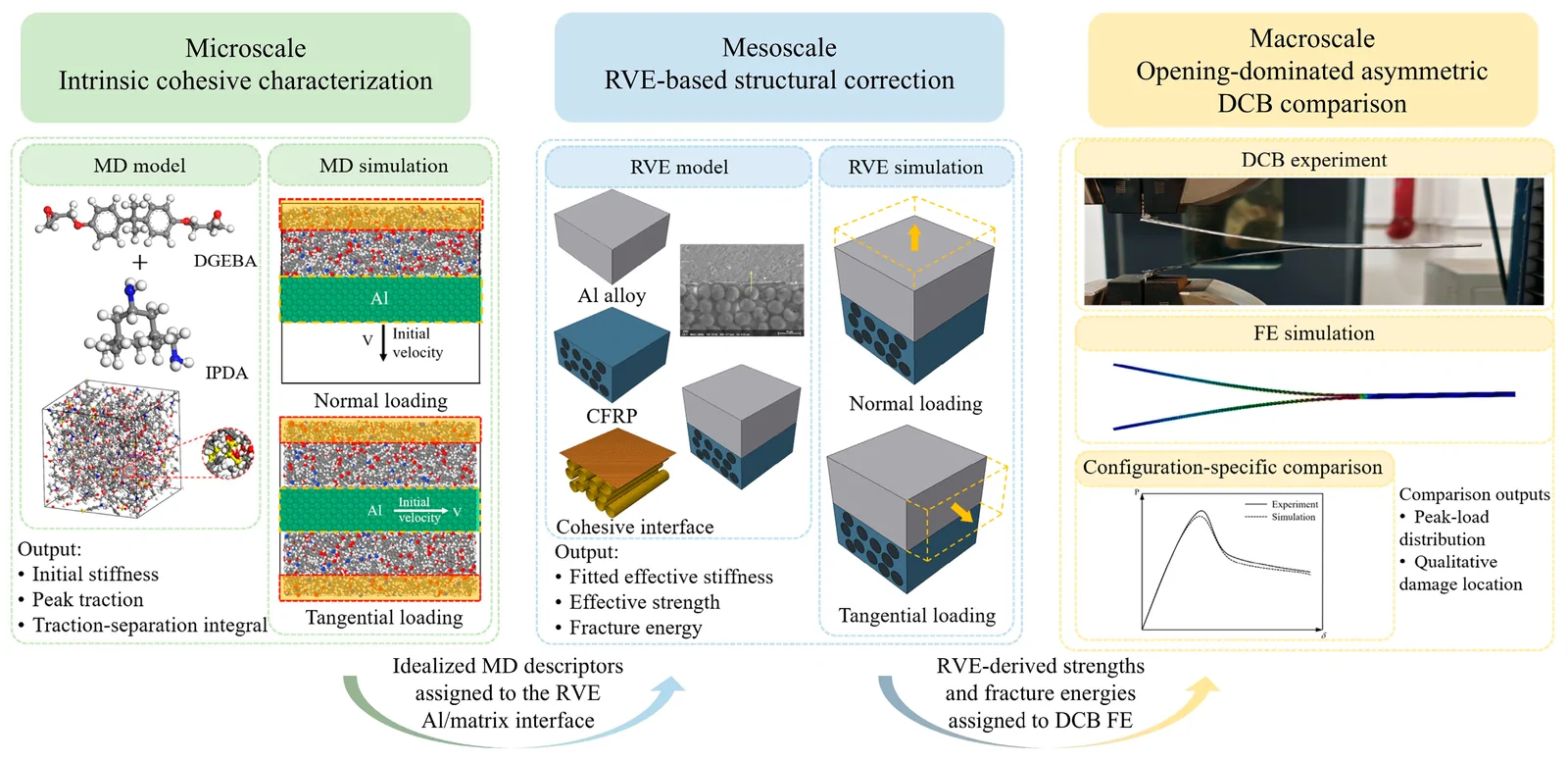
Multiscale MD–RVE–FE framework for configuration-specific transfer of cohesive parameters and model–experiment comparison in CARALL.

The workflow begins with idealized, high-rate Al/epoxy descriptors and converts them through three stochastic RVE realizations (Figure 1). The resulting effective strengths and fracture energies are assigned to an opening-dominated asymmetric DCB model. The three stages are atomistic characterization, configuration-specific mesoscale conversion, and structural model-experiment comparison.

At the atomistic scale, MD simulations resolved the normal-separation and tangential-sliding responses of an ideal, nonbonded Al/epoxy interface at high imposed rates. Initial stiffnesses, peak tractions, and traction-separation integrals were retained as single-realization, model-specific descriptors. After unit conversion, these descriptors parameterized the Al/matrix cohesive interface in the RVE. They were not interpreted as direct properties of the phosphoric-acid-anodized experimental interface or extrapolated to the quasi-static DCB rate.

At the mesoscale, a three-phase RVE represented aluminum, resin, three stochastic carbon-fiber arrangements of the same morphology and coupled Al/matrix and fiber/matrix interfaces; the effective parameters transferred to the structural model are the means over these three realizations. Separate normal and tangential analyses converted the atomistic descriptors into configuration-specific effective parameters for the adopted morphology. Nominal strengths were calculated as the peak reaction forces divided by the 1600 μm^2^ loaded area. Effective stiffnesses were obtained from the fitted initial force-displacement slopes divided by this area, while fracture energies were calculated from the external work divided by the same area. Complete loss of load-carrying capacity defined the endpoint of each response. These outputs apply only to the adopted morphology; they are averages over the three realizations of that morphology and are not ensemble-averaged material properties of the laminate. The fitted RVE stiffnesses were reported as RVE outputs but were not transferred to the structural model.

At the structural scale, only the RVE-derived strengths and fracture energies were assigned directly to the DCB cohesive layer. The isotropic penalty stiffnesses, Knn = Kss = Ktt = 1.0 × 10^5^ MPa/mm, were prescribed numerical inputs rather than quantities derived from the fitted RVE slopes. Assuming in-plane isotropy, the tangential RVE response parameterized both in-plane cohesive components. The structural comparison used the experimental load–displacement responses, peak-load distribution, and qualitative damage location. Equivalent fracture work was excluded because its crack-extension denominator was not traceable and the metric was not equivalent to GIc. The transfer procedure was exercised end to end: the atomistically derived, RVE-corrected parameters were assigned to the structural model without specimen-level inverse fitting. The resulting structural comparison is descriptive and configuration-specific, and the agreement between the predicted and measured responses is limited both by the idealization of the atomistic interface and by the absence of synchronized crack-length and independent fracture-resistance data.

### 2.2. Molecular Dynamics Modeling of the Al/Epoxy Interface

**Figure 2:**
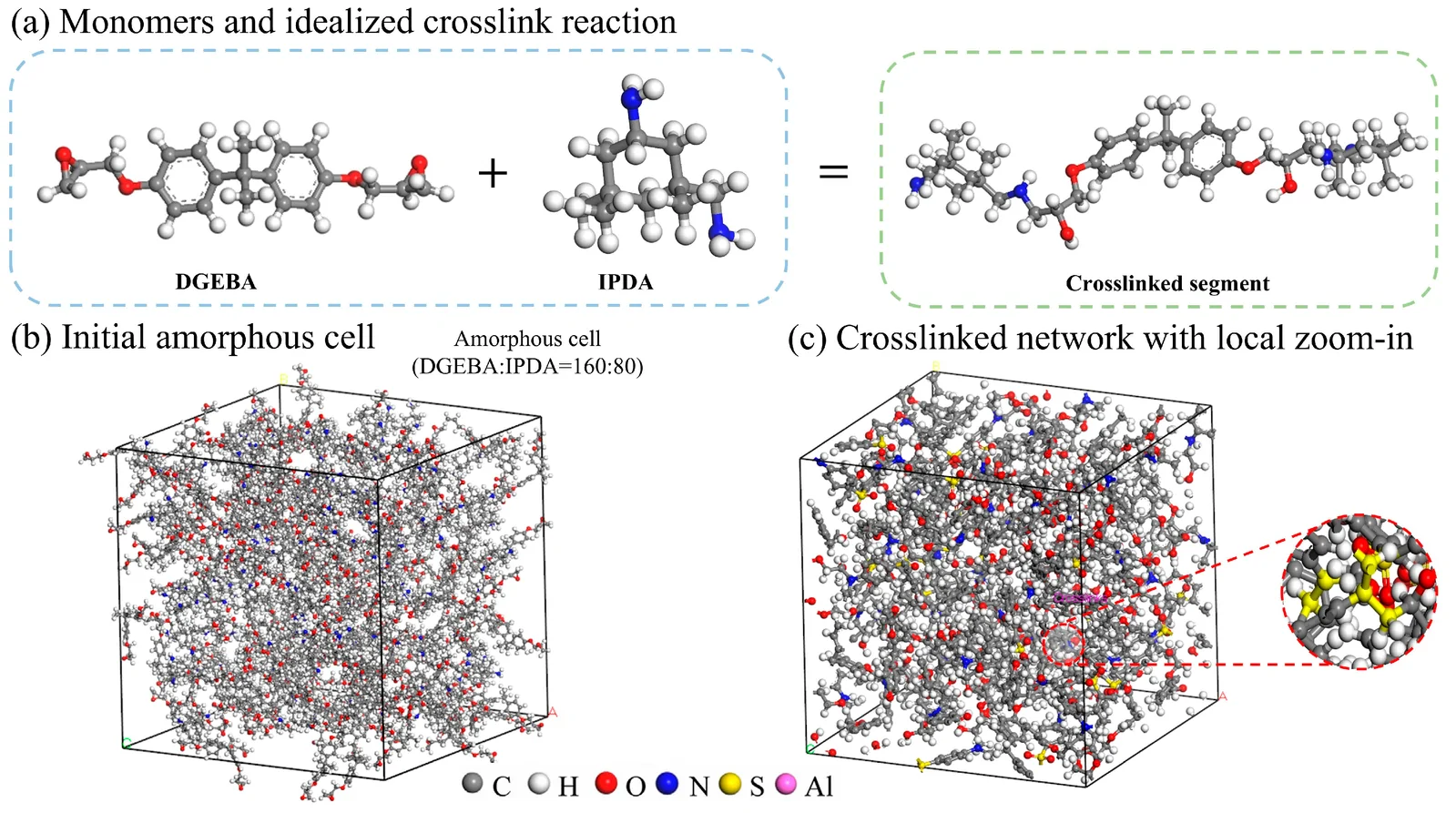
Construction and cross-linking of the DGEBA/IPDA epoxy model: (**a**) monomers and the idealized cross-linking reaction; (**b**) initial amorphous cell (DGEBA:IPDA = 160:80); and (**c**) cross-linked network with a local zoom-in.

**Figure 3:**
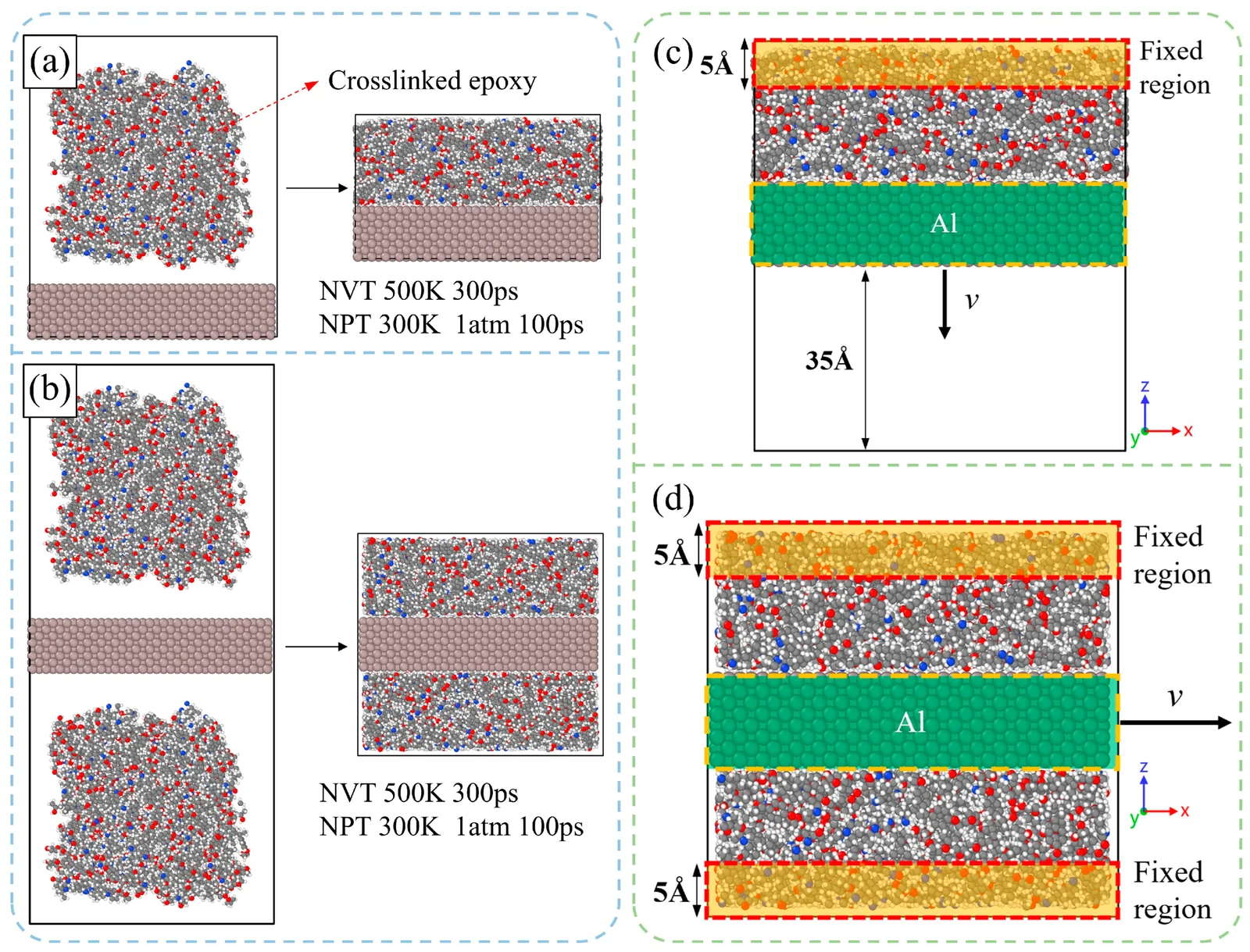
Atomistic Al/epoxy configurations and boundary conditions for (**a**,**c**) normal separation and (**b**,**d**) tangential sliding.

The atomistic simulations generated the Al/epoxy cohesive descriptors required for cross-scale transfer. A cross-linked epoxy network was constructed, and separate interface configurations were prepared for normal separation and tangential sliding (Figure 2 and Figure 3).

The 7901 resin system used as the model epoxy system for the atomistic simulations consisted of diglycidyl ether of bisphenol A (DGEBA; E51) and isophorone diamine (IPDA). The initial amorphous cell contained 160 DGEBA molecules and 80 IPDA molecules at a density of 0.6 g/cm^3^. Following energy minimization, the system was equilibrated for 500 ps in the canonical (NVT) ensemble at 300 K. It was subsequently equilibrated for 500 ps in the isothermal-isobaric (NPT) ensemble at 300 K and 1 atm. The final density was compared with reported values for cured epoxy as an equilibrium check.

Cross-linking was implemented in the Forcite module of BIOVIA Materials Studio 2021 (Dassault Systèmes, San Diego, CA, USA) using a stepwise Perl algorithm. Proximity searches increased the cutoff distance from 4.0 to 12.0 Å in 0.5 Å increments, with no more than three bonding attempts at each cutoff. A smoothing procedure reduced local distortion before covalent bond formation. The target bond length was tightened to 1.47 Å in three stages, each followed by structural relaxation. Cross-linking used a 0.2 fs time step, the Fine accuracy setting, an Andersen thermostat, and atom-based charges. The final network contained approximately 13,200 atoms and achieved a cross-linking degree of 80%. Its dimensions were 4.71 × 4.71 × 4.71 nm^3^, and its density was 1.08 g/cm^3^.

The aluminum substrate was modeled as an atomically flat Al(111) surface governed by an embedded-atom method potential. PCFF described the epoxy, and a Lennard-Jones 12-6 potential described only the nonbonded Al/epoxy interactions. The model contained no native oxide or Al_2_O_3_, surface hydroxyl groups, roughness, defects, or interfacial covalent or coordination bonds. It therefore provides an idealized, physically adsorbed reference interface rather than a chemical or structural replica of the phosphoric-acid-anodized 5052 aluminum surface. Velocity-Verlet integration was used throughout, with NVT and NPT ensembles applied during relaxation and densification. Periodic boundary conditions were imposed in the in-plane directions to reduce finite-size effects.

For normal separation, a 35 Å vacuum layer prevented periodic-image interactions along the loading direction. The uppermost 5 Å of epoxy was fixed, and periodicity was retained only in the in-plane directions. Following 200 ps of NVT relaxation at 300 K, the aluminum layer was translated normal to the interface at the imposed rates described below (Figure 3a,c). For tangential sliding, the model was relaxed for 200 ps in the NVT ensemble at 300 K. The outermost 5 Å of both epoxy blocks was constrained, and the aluminum layer was translated parallel to the interface at the same imposed rates.

To evaluate sensitivity to the imposed MD loading rate, the normal-separation and tangential-sliding simulations were conducted at 0.001, 0.002, 0.003, 0.004, and 0.005 Å/fs. The same initial configurations, equilibration procedure, time step, boundary conditions, loading endpoint, integration interval, and postprocessing method were used in all five cases. The descriptor set associated with the 0.005 Å/fs calculation was used in the subsequent RVE analysis, while the remaining cases were used to assess rate sensitivity. These calculations characterize sensitivity within the accessible high-rate MD window and do not provide an extrapolation to the quasi-static DCB rate. To evaluate sensitivity to the Al/epoxy interaction strength, the Lennard-Jones ε parameter of all Al–epoxy cross interactions was additionally varied by factors of 0.5 and 1.5 at the 0.005 Å/fs loading rate, with the initial configuration, equilibration procedure, time step, boundary conditions, loading endpoint, integration interval, and postprocessing method identical to the baseline calculation.

The normal-separation and tangential-sliding configurations were constructed from the same cross-linked epoxy network. Only one atomistic realization was analyzed. The five imposed rates were evaluated using this same realization, so the rate study did not constitute independent configuration sampling. Independently packed and cross-linked networks and atomistic cell-size variants were not evaluated. Accordingly, the extracted peak tractions and traction-separation integrals are single-realization, high-rate, model-specific descriptors rather than ensemble-averaged or quasi-static material properties.

For tangential sliding, the aluminum layer was enclosed by cross-linked epoxy to form a symmetric resin/Al/resin configuration (Figure 3b,d). This symmetric arrangement minimized bending and rotation of the aluminum layer during relative sliding. The constrained aluminum layer was gradually brought into contact with both epoxy blocks. The assembled system was then relaxed for 100 ps under NPT conditions at 300 K and 1 atm.

### 2.3. Mesoscale RVE Modeling of the CARALL Interlaminar Region

The mesoscale RVE transformed the idealized Al/epoxy response into effective cohesive parameters for the local CARALL interlaminar region. It was formulated as a physically informed local cell rather than a statistical representation of the entire laminate. The model captured heterogeneity governing load transfer, stress redistribution, and interactions between Al/matrix and fiber/matrix damage.

A 40 × 40 × 40 μm^3^ RVE was constructed based on the observed interlaminar geometry. The fibers were aligned with and extended through the X direction. Its 40 × 40 μm^2^ transverse cross-section contained 18 nonoverlapping cylindrical fibers with a diameter of 7 μm, giving a geometric fiber volume fraction of 18π(3.5 μm)^2^/(40 μm)^2^ = 43.3% and a transverse span of 5.71 fiber diameters. This cell size captures the local interlaminar morphology while remaining computationally tractable.

**Figure 4:**
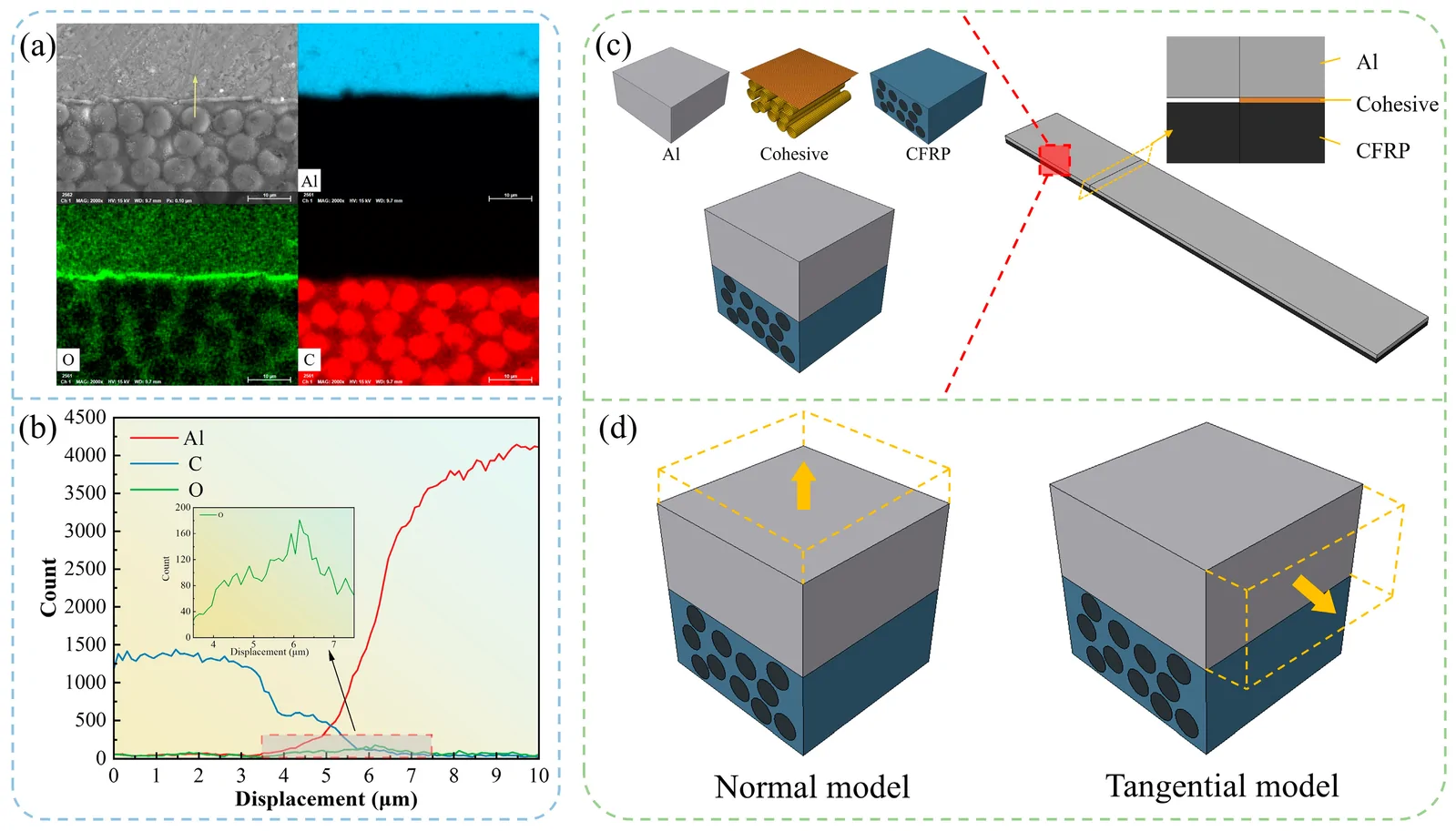
Experimental characterization and mesoscale representation of the CARALL interlaminar region: (**a**) SEM/EDS maps; (**b**) elemental line profiles; (**c**) RVE construction; and (**d**) normal and tangential loading configurations.

Scanning electron microscopy (SEM; Sigma 560, Carl Zeiss Microscopy GmbH, Jena, Germany) and energy-dispersive X-ray spectroscopy (EDS; Oxford Instruments NanoAnalysis, High Wycombe, UK) informed the interfacial geometry (Figure 4). Aluminum was adjacent to CFRP, with fiber cross-sections embedded in the resin matrix. Spatial gradients in Al, C, and O suggested an interfacial transition region approximately 3 μm thick. To reduce computational cost, this finite-thickness region was represented by zero-thickness cohesive interfaces.

Two cohesive-interface families represented distinct mechanical roles within the RVE. The Al/matrix interface transmitted the primary load across the interlaminar region. The surrounding fiber/matrix interfaces represented local debonding, matrix constraint, and stress redistribution near the Al/matrix interface. Including both interface families allowed damage at the two interface levels to interact during loading.

The 5052 aluminum and epoxy matrix were modeled as isotropic linear-elastic materials, whereas the T700 fibers were modeled as transversely isotropic (Table 1). The Al/matrix cohesive parameters were obtained from the MD calculations. Fiber/matrix cohesive parameters were adopted from a comparable T700/epoxy study [69] and are listed in Table 2. These literature-derived values were used as fixed constitutive inputs for the present resin system.

**Figure 5:**
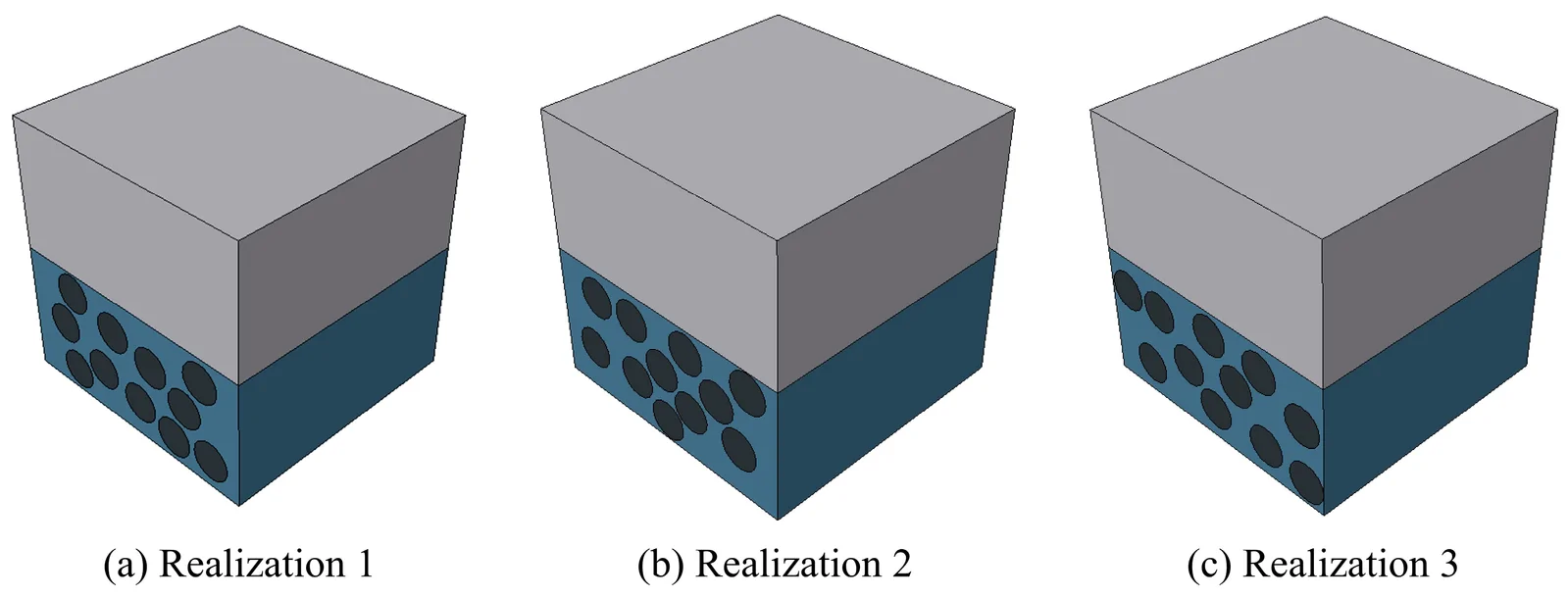
Three independent stochastic fiber arrangements of the same morphology used in the mesoscale RVE.

A 40 × 40 × 40 μm^3^ RVE was discretized using C3D8R elements for the aluminum, epoxy matrix, and fibers and COH3D8 elements for the Al/matrix and fiber/matrix interfaces. Three independent stochastic fiber arrangements of the same morphology were generated and analyzed under identical loading and postprocessing conditions (Figure 5), and the RVE was additionally discretized with element sizes of 0.5, 0.8, and 1.2 μm; the mesh-sensitivity study was performed for a single realization, whereas the realization-to-realization variability is reported separately in Section 3.2. Both interfaces followed a bilinear traction-separation law. Damage initiation was governed by the quadratic nominal-stress criterion, and energy-based damage evolution used linear softening. Mixed-mode evolution followed the Benzeggagh-Kenane relation with η = 1.5 and GIIIc = GIIc. Complete failure corresponded to SDEG = 1, at which point cohesive-element deletion was enabled. A viscous-regularization coefficient of 1.0 × 10^−4^ was applied.

The Al/matrix interface was parallel to the X-Z plane, Y was the interface-normal direction, and the fiber direction was X. Matching nodes on opposing X and Z faces were linked by periodic constraint equations of the form $u_{i}^{+}$ − $u_{i}^{-}$ = *H*_*ij*_($x_{j}^{+}$ − $x_{j}^{-}$), where *H* is the prescribed macroscopic displacement gradient. The CFRP-side bottom surface was fixed to suppress rigid-body motion, and the aluminum-side loading surface was coupled to a reference point. Normal loading was imposed through U2 to a total displacement of 1.0 μm, and the corresponding RF2 was extracted. Tangential loading was imposed through U1 to a total displacement of 1.5 μm, and RF1 was extracted. The nominal traction was calculated as the total reaction force divided by the 1600 μm^2^ loaded area.

The RVE and DCB models were solved using Abaqus/Explicit 2021 (Dassault Systèmes Simulia Corp., Providence, RI, USA) with displacement-controlled smooth-step amplitudes. In the RVE calculations, semi-automatic mass scaling was applied to the whole model at the beginning of the analysis with a target stable time increment of 1.0 × 10^−6^ s.

### 2.4. Aluminum Surface Treatment, Specimen Fabrication, and Opening-Dominated Asymmetric DCB Testing

**Figure 6:**
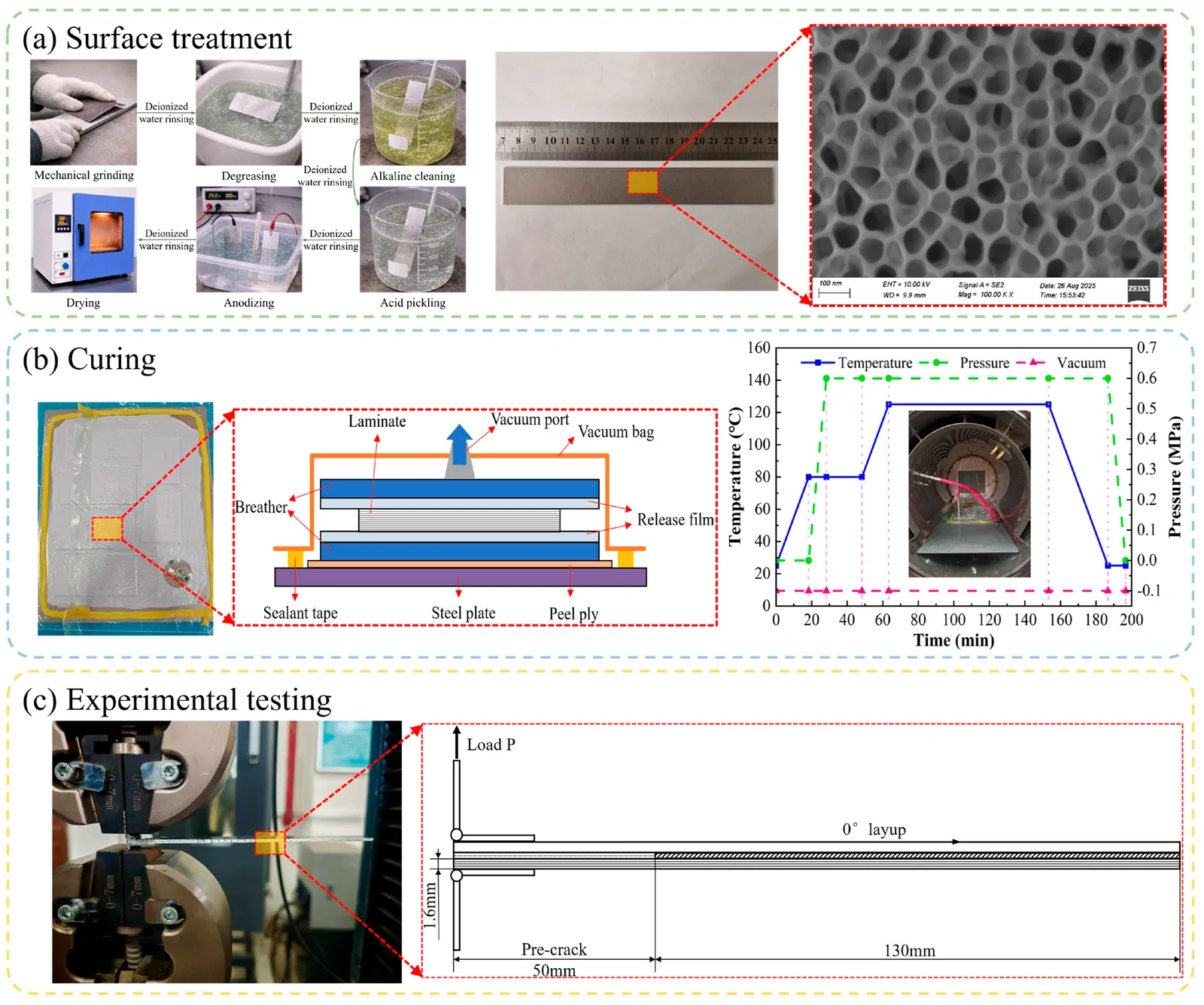
Aluminum surface treatment, CARALL specimen fabrication, and asymmetric DCB testing: (**a**) surface pretreatment; (**b**) vacuum-assisted hot-press co-curing; and (**c**) opening-dominated asymmetric DCB test configuration.

CARALL specimens were fabricated from 5052 aluminum sheets (Henan Mingtai Al. Industrial Co., Ltd., Gongyi, China) and T700 unidirectional carbon-fiber prepreg (Weihai Guangwei Composites Co., Ltd., Weihai, China). Before layup, the aluminum surfaces were sequentially ground, ultrasonically cleaned (SAY-10L ultrasonic cleaner, Suzhou Shengaoyuan Environmental Protection Technology Co., Ltd., Suzhou, China), degreased, alkaline-cleaned, acid-cleaned, anodized, and dried (Figure 6). Each treatment step was followed by rinsing with deionized water. The alkaline- and acid-cleaning baths were prepared using 100 g of NaOH and 100 g of HNO_3_ (both from Xilong Scientific Co., Ltd., Shantou, China), respectively, and both treatments were conducted at room temperature. During anodizing, the aluminum sheet served as the anode and a lead plate served as the cathode. A voltage of 25 V was applied for 10 min using an MSP6200 DC power supply (Suzhou Meiensi Electronic Technology Co., Ltd., Suzhou, China) in an electrolyte prepared using 100 g of H_3_PO_4_ (Xilong Scientific Co., Ltd., Shantou, China) and 1–3 g of sodium gluconate (Sinopharm Chemical Reagent Co., Ltd., Shanghai, China) at 25 ± 3 °C. The resulting porous morphology supported resin infiltration and mechanical interlocking at the Al/epoxy interface.

Plan-view SEM revealed an open porous surface morphology after anodizing (Figure 6a), and cross-sectional SEM/EDS showed the oxide-related interfacial region and the distributions of Al, O, and C (Figure 4a). Although the present images do not resolve resin penetration into individual pores or identify surface hydroxylation and specific interfacial bonds, the observed porous morphology supports resin infiltration and mechanical interlocking at the interface.

Four DCB specimens (n = 4) were manufactured by vacuum-assisted hot-press co-curing in a KR-YZ vacuum hot press (Dongguan Kerui Instrument Technology Co., Ltd., Dongguan, China), using ASTM D5528 [71] as the specimen and loading framework. Each specimen measured 180 × 25 × 3.2 mm and had a single-sided [Al/0°_16_] layup comprising one aluminum sheet and 16 T700 prepreg plies. Two 0.05-mm-thick PTFE films (Nantong Longjin Membrane Technology Co., Ltd., Nantong, China) were inserted between the aluminum sheet and the first prepreg ply to produce a 50-mm starter crack. A steel support was used to maintain specimen flatness during layup. Peel ply, release film, breather cloth, and a vacuum bag completed the vacuum assembly and facilitated demolding. A gauge vacuum pressure of −0.1 MPa was maintained throughout specimen preparation.

A two-stage curing cycle was employed to limit residual thermal stresses. The assembly was first heated to 80 °C at 3 °C/min and held for 30 min to promote resin flow, interfacial wetting, and air removal. A pressure of 0.6 MPa was then applied, after which the temperature was increased to 125 °C at 3 °C/min and held for 90 min. The laminate was subsequently cooled at the same rate. This curing schedule promoted laminate consolidation and bonding across the Al/epoxy interlaminar region.

The DCB specimens and loading arrangement followed ASTM D5528 [71] as the experimental framework. The specimens were loaded through bonded steel piano hinges at a crosshead rate of 1 mm/min using a KDW-10 electronic universal testing machine (Jinan Kaide Instrument Co., Ltd., Jinan, China), and crack propagation along the Al/matrix interfacial region was monitored. Crack length was tracked qualitatively rather than recorded quantitatively at synchronized load–displacement points, so the retained data support the load–displacement response and the qualitative damage location but not compliance calibration, ASTM GI values, or R-curves. Because the single-sided [Al/0°16] configuration comprises dissimilar aluminum and composite arms with unequal flexural rigidities, ASTM D5528 serves here as a specimen and loading framework for this opening-dominated asymmetric DCB configuration.

### 2.5. Macroscale Finite-Element Modeling of the Asymmetric DCB Specimen

A three-dimensional finite-element model of the opening-dominated asymmetric DCB configuration was developed in Abaqus/Explicit. The RVE-derived cohesive strengths and fracture energies were assigned directly to the structural interface without fitting to the experimental load–displacement response. The isotropic fiber/matrix interface penalty stiffness (K*_nn_* = K*_ss_* = K*_tt_* = 1.0 × 10^5^ MPa/mm) was prescribed numerically [69]. The model was used to assess the transferred parameter set for the reported specimen configuration.

The experimental specimen comprised one nominally 1.6-mm-thick 5052 aluminum arm and a composite arm manufactured from sixteen nominally 0.10-mm T700 prepreg plies. The sixteen plies were represented in the FE model as a homogenized 1.6-mm orthotropic continuum. A 0.01-mm COH3D8 layer numerically represented the interlaminar region. The nominal experimental specimen thickness was 3.2 mm, and the specimen length and width were 180 and 25 mm, respectively.

The aluminum and composite arms were discretized using C3D8R elements. The nominal in-plane element size was 1.0 mm in the traction-free precracked region and refined to 0.5 mm in the bonded region ahead of the crack tip. To assess mesh sensitivity, the bonded-region element size was varied from 0.5 to 1.0 mm while all material, loading, and mass-scaling settings were held fixed. The structural penalty stiffness was varied by factors of 0.5 and 2, and the transferred cohesive strength and cohesive fracture energy were each varied by factors of 0.8 and 1.2, i.e., within a prescribed ±20% band around the transferred values, to characterize the sensitivity of the predicted response over that band. This band is prescribed for the present local sensitivity analysis rather than derived from a measured or statistically sampled distribution, and the resulting variations are therefore not treated as a quantified uncertainty range for the transferred parameters. Within each perturbation, the normal and tangential strengths were scaled simultaneously by the same factor and the Mode I and Mode II fracture energies were likewise scaled simultaneously by the same factor; the two parameter families were perturbed one at a time. The kinetic-to-internal-energy ratio was monitored in each case to confirm quasi-static conditions. The 50-mm PTFE starter crack was represented by a traction-free interface segment, while the remaining interface contained COH3D8 elements governed by the bilinear cohesive law. The composite arm was represented as an orthotropic elastic continuum using the material properties listed in Table 3.

The upper and lower hinge-loading regions were connected to RP-Top and RP-Bot using distributing couplings. RP-Bot was constrained in U1, U2, and U3. At RP-Top, U1 and U3 were constrained and an opening displacement of 45 mm was applied in U2 using a smooth-step amplitude; the rotational degrees of freedom remained free. Geometric nonlinearity was enabled. The numerical load–displacement response was obtained from RF2 and U2 at RP-Top. For the DCB model, a mass-scaling factor of 1.0 × 10^−3^ was applied.

The numerical results were compared with the measured load–displacement responses, peak loads, and observed damage location to assess the overall structural response of the asymmetric DCB specimen.

## 3. Results and Discussion

### 3.1. Model-Specific Response of the Idealized Al/Epoxy Interface

The relaxed idealized Al/epoxy interface exhibited a binding energy of −683.88 kcal/mol. Because the atomically flat Al(111) surface contained no oxide, hydroxyl groups, roughness, defects, or interfacial chemical bonds, the calculated adhesion originated solely from nonbonded interactions. By contrast, the experimental interface contained a porous, oxide-related surface structure, as shown in Figure 4a and Figure 6a, and previous studies have shown that oxide chemistry and surface functional groups affect epoxy/aluminum adhesion [27,28,29]. These chemical and morphological mechanisms may influence the peak traction, traction–separation integral, and curve shape. The MD outputs are therefore interpreted as model-specific descriptors of the idealized, nonbonded interface. 

Five imposed rates from 0.001 to 0.005 Å/fs were evaluated while all other simulation and postprocessing conditions were held constant. The normal peak traction increased from 443.76 to 470.09 MPa, and the tangential peak traction increased from 333.52 to 352.93 MPa, corresponding to changes of 5.93% and 5.82%, respectively. The Mode I and Mode II traction–separation integrals varied nonmonotonically across the tested high-rate interval (Table 4), so the peak tractions showed limited variation whereas the energy descriptors exhibited greater rate sensitivity. Because all tested MD rates remain far above the DCB rate, the descriptors are interpreted within the accessible high-rate MD window. To assess whether the transferred descriptors are controlled by the idealized Al/epoxy interaction, the Lennard-Jones interaction strength ε between the aluminum and epoxy atoms was varied by factors of 0.5 and 1.5 while the configuration, equilibration, loading rate (0.005 Å/fs), and postprocessing were held fixed. Relative to the baseline value, the normal peak traction decreased by 60% at 0.5ε and increased by 55% at 1.5ε, whereas the tangential peak traction decreased by 28% and increased by 25% (Table 5). The corresponding works of separation changed in the same direction. The normal response is therefore more sensitive to the interaction strength than the tangential response. Because these idealized-interface descriptors are transferred to the RVE and then to the structural model, the results confirm that the transferred parameters are conditional on the assumed nonbonded Al/epoxy interaction and should not be regarded as intrinsic properties of the phosphoric-acid-anodized experimental interface.

**Figure 7:**
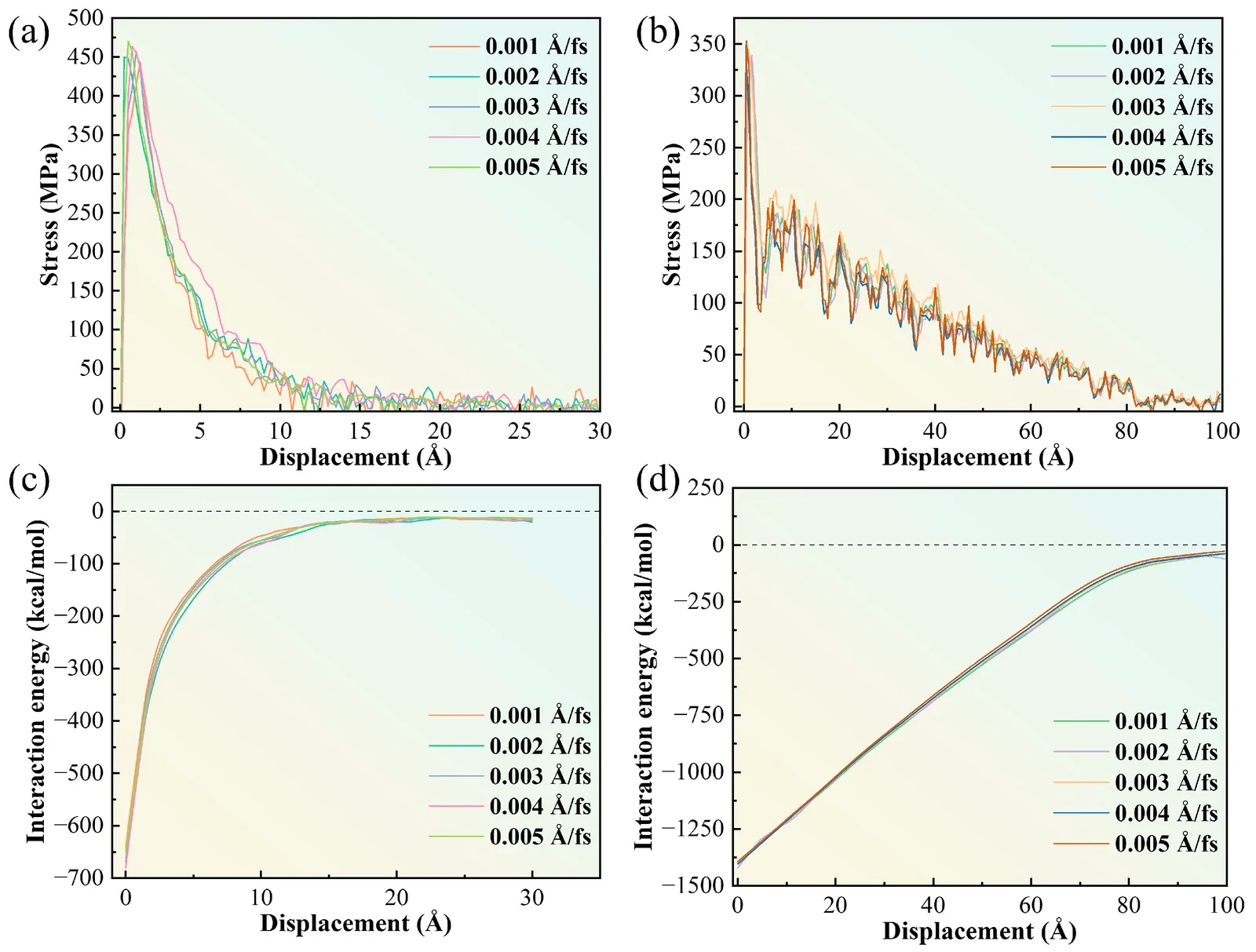
Rate-dependent responses of the idealized Al(111)/epoxy interface at imposed loading rates of 0.001–0.005 Å/fs: (**a**) normal traction–separation curves; (**b**) tangential traction–sliding curves; (**c**) normal interaction-energy curves; and (**d**) tangential interaction-energy curves.

**Figure 8:**
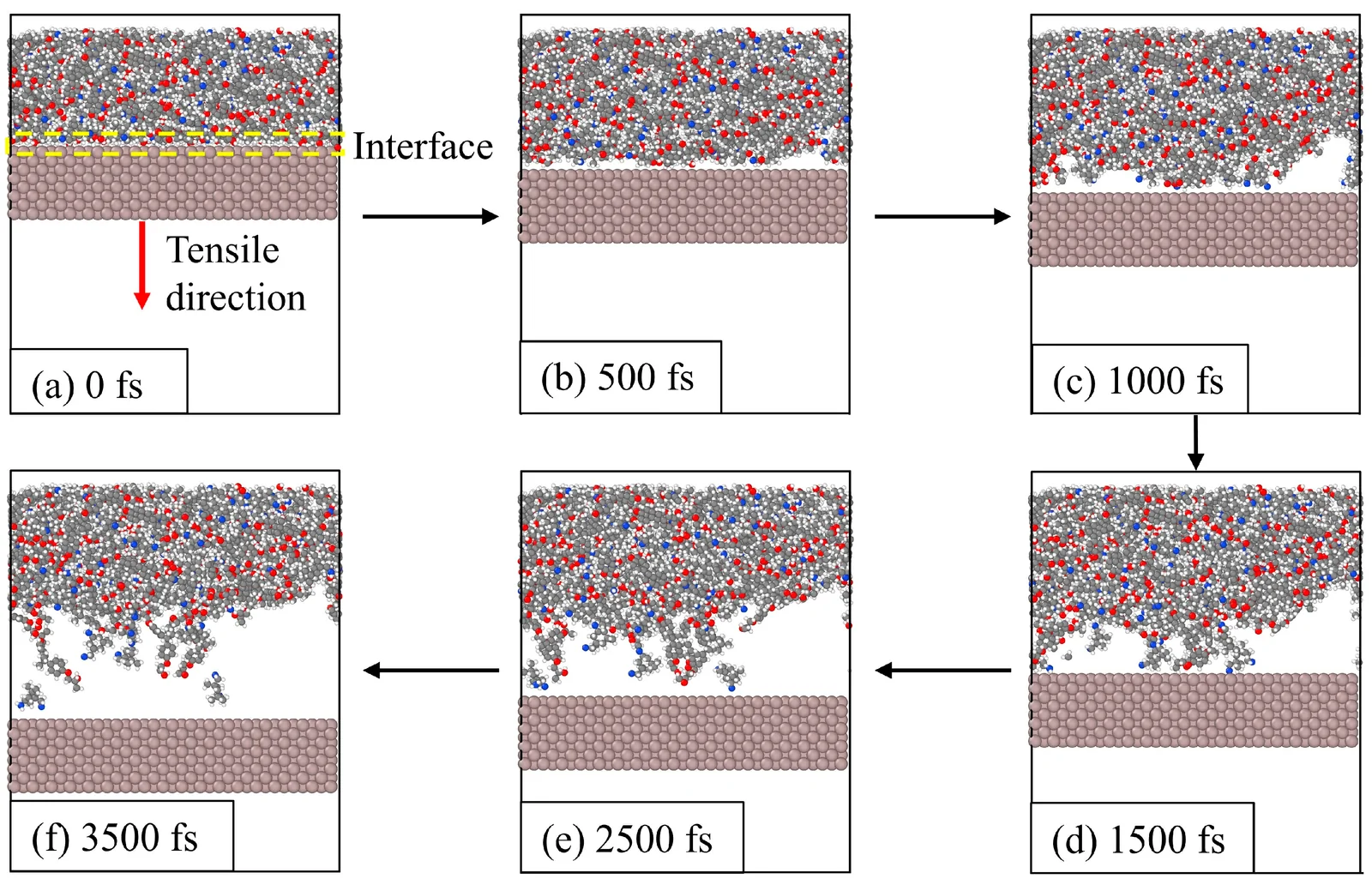
Atomistic configurations of the Al/epoxy interface during normal separation at an imposed rate of 0.005 Å/fs. The atom colours are as defined in Figure 2.

Under normal separation at the selected rate of 0.005 Å/fs, the traction reached 470.09 MPa and then decreased rapidly (Figure 7a). It approached zero at approximately 11 Å, indicating near-complete loss of interfacial traction. The atomistic configurations showed progressive interface separation, limited molecular-chain pullout, and a largely intact epoxy network (Figure 8). The concurrent reduction in interaction-energy magnitude tracked the progressive weakening of nonbonded interfacial attraction.

**Figure 9:**
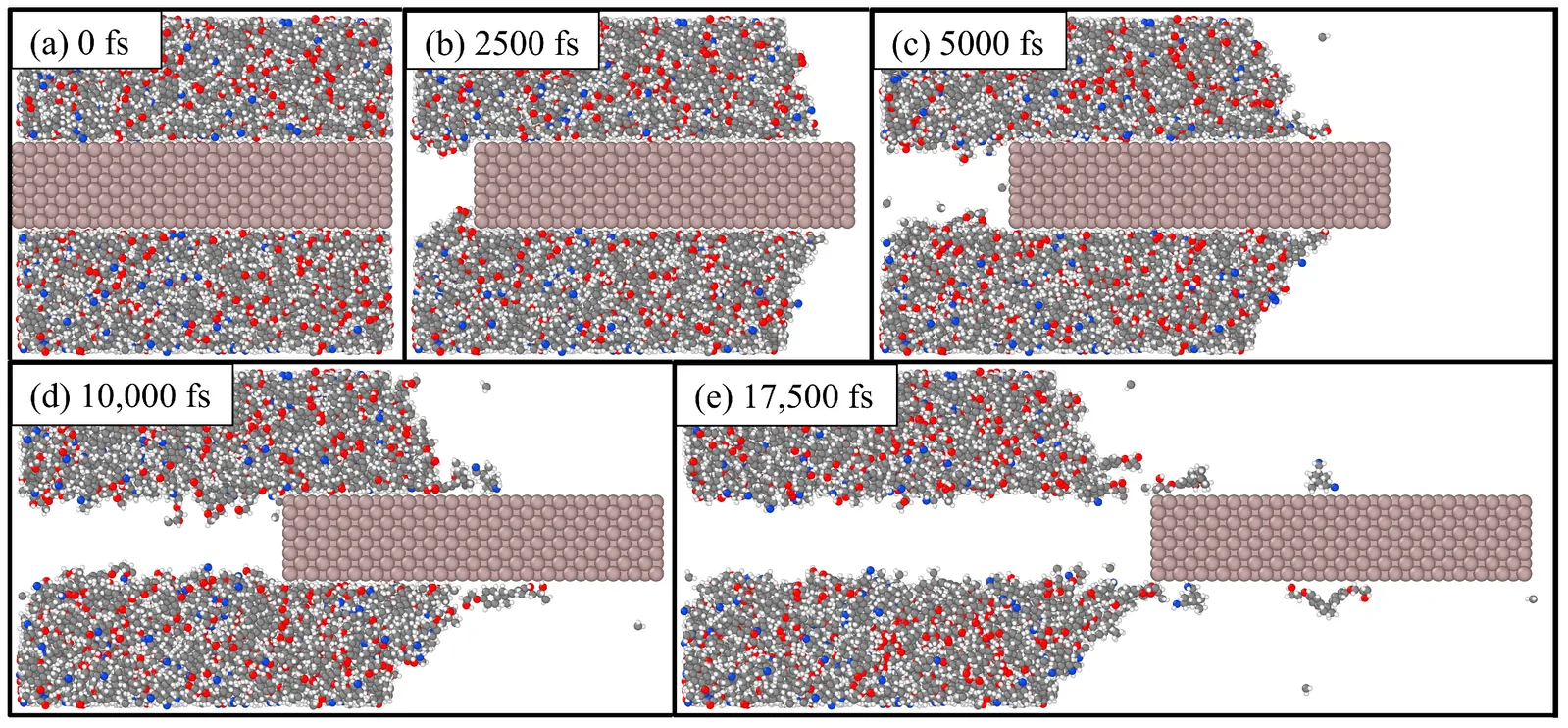
Atomistic configurations of the Al/epoxy interface during tangential sliding at an imposed rate of 0.005 Å/fs. The atom colours are as defined in Figure 2.

At the selected rate of 0.005 Å/fs, tangential sliding produced a peak traction of 352.93 MPa, 24.9% below the normal peak. The postpeak response extended to approximately 65 Å and showed pronounced fluctuations (Figure 7b), accompanied by molecular rearrangement, contact loss, and intermittent residual contacts (Figure 9). This longer postpeak response yielded a larger traction–separation integral than normal separation, reflecting greater energy dissipation under tangential sliding.

Model-specific cohesive descriptors were extracted from the MD traction–separation curves. The peak tractions defined the normal and tangential damage-initiation strengths:

(1) $$t_{n,0}^{MD}=\underset{\delta_{n}}{\max}\left[t_{n}\left(\delta_{n}\right)\right],\;t_{s,0}^{MD}=t_{t,0}^{MD}=\underset{\delta_{s}}{\max}\left[t_{s}\left(\delta_{s}\right)\right]$$

The initial slopes of the traction–separation curves defined the corresponding penalty stiffnesses:

(2) $$K_{nn}^{MD}={\frac{dt_{n}}{d\delta_{n}}|}_{\delta_{n}\to 0},K_{ss}^{MD}=K_{tt}^{MD}={\frac{dt_{s}}{d\delta_{s}}|}_{\delta_{s}\to 0}$$

The areas under the normal and tangential traction–separation curves defined the Mode I and Mode II fracture energies:

(3) $$G_{Ic}^{MD}=\int_{0}^{\delta_{n,f}}t_{n}\;d\delta_{n},G_{IIc}^{MD}=\int_{0}^{\delta_{s,f}}t_{s}\;d\delta_{s}$$

Here, *t*, *δ*, *K*, and *G* denote traction, separation, penalty stiffness, and fracture energy, respectively. Subscripts *n*, *s*, and *t* identify the normal and two in-plane tangential directions. Subscript *f* denotes complete traction loss, while superscript MD identifies the atomistic output. The *s*- and *t*-direction properties were assigned equal values based on the in-plane isotropy assumption.

The MD descriptors were subsequently converted to the micrometer-based unit system used in the mesoscale RVE. The strength values remained unchanged because stress is independent of the selected length unit:

(4) $$t_{0}^{RVE}=t_{0}^{MD}$$

Because 1 μm = 10^4^ Å, fracture energy and penalty stiffness were converted as follows:

(5) $$G^{RVE}=10^{-4}G^{MD},K^{RVE}=10^{4}K^{MD}$$

In Equation (5), *G*^MD^ and *G*^RVE^ are expressed in MPa Å and μN/μm, respectively. Similarly, *K*^MD^ and *K*^RVE^ are expressed in MPa/Å and MPa/μm, respectively.

The extracted normal and tangential strengths were 470.09 and 352.93 MPa, respectively (Table 6). The Mode I and Mode II fracture energies were 1778.71 and 7373.55 MPa Å, corresponding to 0.1778 and 0.7373 μN/μm. The Mode II fracture energy was therefore 4.15 times the Mode I value. The normal and tangential stiffnesses were 940.16 and 235.29 MPa/Å, corresponding to 9.4016 × 10^6^ MPa/μm and 2.3529 × 10^6^ MPa/μm. Table 6 summarizes these descriptors and their converted values for transfer to the mesoscale RVE. Because the interlaminar response cannot be measured directly at this scale, the descriptors must be converted through a mesoscale representation before they can be compared with the structural test; the absence of fitting to the DCB response therefore establishes that the transfer is exercised without specimen-level calibration, and not that the resulting structural comparison validates the descriptors.

### 3.2. Mesoscale Damage Evolution and Configuration-Specific Effective Interfacial Properties

**Figure 10:**
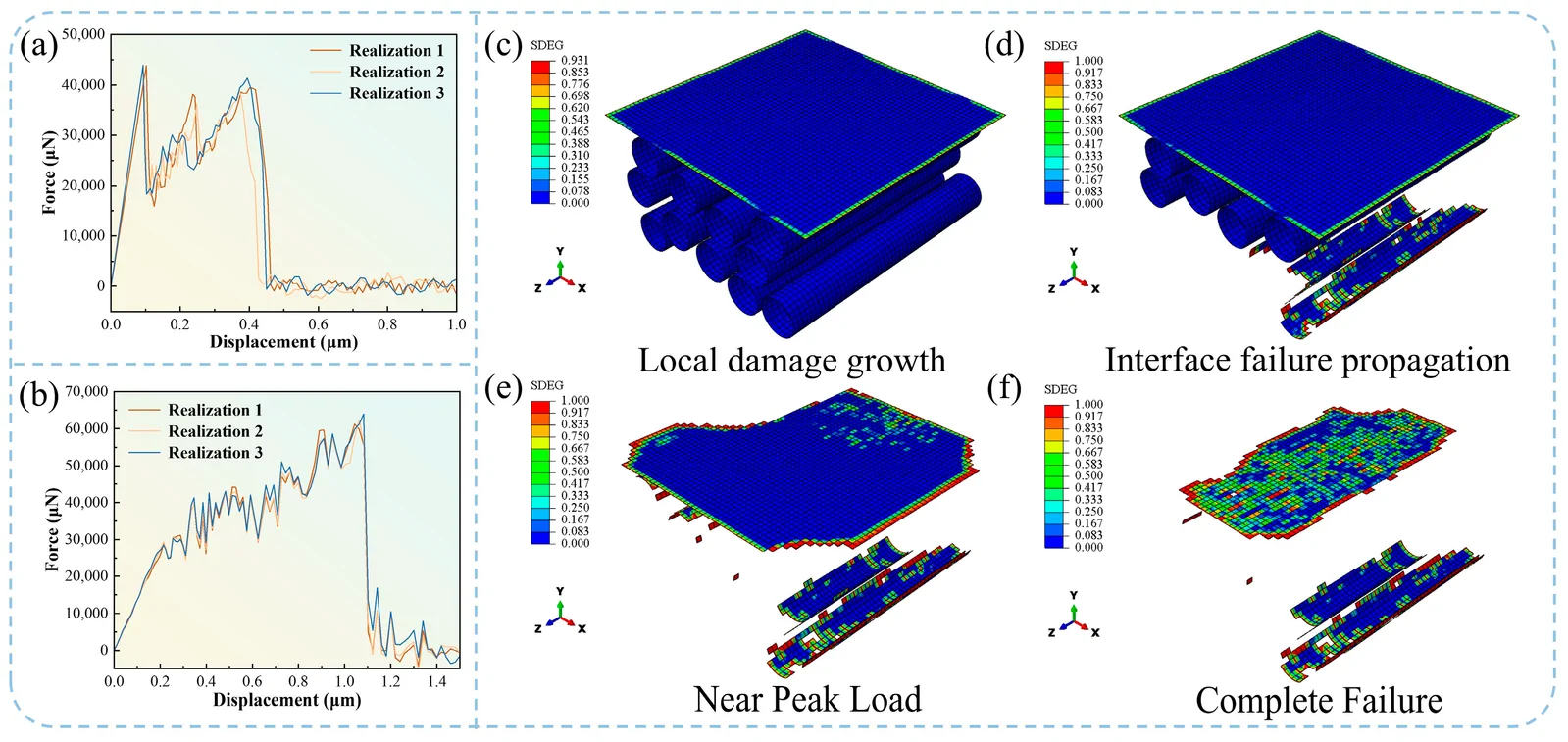
Force–displacement responses and damage evolution for three stochastic fiber realizations: (**a**) normal responses; (**b**) tangential responses; and (**c**–**f**) progressive damage at the fiber/matrix and Al/matrix interfaces of a representative realization.

The mesoscale RVE quantified how the local morphology modified the intrinsic Al/epoxy response before transfer to the structural model. The RVE had dimensions of 40 × 40 × 40 μm^3^ and incorporated the Al substrate, epoxy matrix, reinforcing fibers, and the Al/matrix and fiber/matrix interfaces. Normal and tangential displacement-controlled loading was applied to obtain the force–displacement responses and damage evolution. Three stochastic fiber realizations of the same morphology were compared (Figure 10), and the fiber/matrix-to-Al/matrix damage sequence persisted in all three. Their force–displacement responses closely agreed in both loading modes: the between-realization coefficients of variation were 1.20% for the normal strength, 1.66% for the tangential strength, 4.29% for the Mode I fracture energy, and 4.45% for the Mode II fracture energy, and the individual values together with their means and standard deviations are listed in Table 7. The mean effective normal and tangential strengths were 27.42 ± 0.33 and 39.04 ± 0.65 MPa, and the mean Mode I and Mode II fracture energies were 0.36 ± 0.02 and 0.81 ± 0.04 N/mm, respectively; these means were transferred to the structural model.

Mesh convergence was assessed separately for a single realization by varying the element size from 0.5 to 1.2 μm (Table 8); the effective strengths varied by less than 5% and the fracture energies by less than 14% with respect to the discretization. The between-realization spread and the mesh sensitivity are therefore distinct quantities, and the values reported in Table 7 characterize the adopted morphology and discretization rather than the material system as a whole.

Under normal separation, the reaction force initially increased almost linearly and reached a peak of 43,887.4 μN. Damage first developed at the fiber/matrix interfaces and subsequently extended through neighboring interfacial regions as deformation and local load redistribution progressed. The post-peak force reduction was accompanied by progressive damage at both the fiber/matrix and Al/matrix interfaces. This sequence describes the damage progression in the adopted RVE realizations.

Under tangential loading, the reaction force similarly exhibited an approximately linear initial response before reaching a peak of 62,432.0 μN. Progressive fiber/matrix and Al/matrix interfacial damage was observed during the subsequent nonlinear and softening stages. The different normal and tangential responses demonstrate the loading-mode dependence of the effective behavior.

The initial stiffnesses were determined by ordinary least-squares linear regression over the linear portions of the force–displacement curves. For normal loading, fitting over 0–0.01959 μm gave dF/dδ = 4.73826 × 10^5^ μN/μm, with R^2^ = 0.9999995. After normalization by the 40 × 40 μm^2^ loaded area, the fitted normal stiffness was K*_nn_*_, RVE_ = 2.9614 × 10^5^ MPa/mm. For tangential loading, fitting over 0–0.19637 μm gave dF/dδ = 1.36726 × 10^5^ μN/μm, with R^2^ = 0.99999998, corresponding to K*_ss_*_, RVE_ = K*_tt_*_, RVE_ = 8.5454 × 10^4^ MPa/mm.

These fitted stiffnesses are RVE outputs and were not used as the structural penalty stiffnesses. In the structural cohesive-zone model, K*_nn_* = K*_ss_* = K*_tt_* = 1.0 × 10^5^ MPa/mm was retained as the prescribed penalty stiffness. The prescribed value is 66.2% lower than the RVE-fitted normal stiffness and 17.0% higher than the RVE-fitted tangential stiffness.

The nominal normal and tangential tractions were obtained by normalizing the reaction forces by the loaded area *A*:

(6) $$t_{n}=\frac{F_{n}}{A},t_{s}=\frac{F_{s}}{A}.$$

The effective strengths were defined as the maxima of the corresponding traction–separation curves. The Mode I and Mode II fracture energies were calculated from the area-normalized work of separation:

(7) $$G_{I}=\frac{1}{A}\int F_{n}\;d\delta_{n},G_{II}=\frac{1}{A}\int F_{s}\;d\delta_{s}.$$

Using these definitions, the three realizations yielded mean effective normal and tangential strengths of 27.42 and 39.04 MPa, respectively, together with mean Mode I and Mode II fracture energies of 0.36 and 0.81 N/mm (Table 9). Relative to the intrinsic Al/epoxy strengths used as cohesive inputs, the corresponding reductions in mean strength were approximately 94.2% and 88.9%. These percentages quantify the attenuation for the present RVE configuration. The reduction in effective strength occurred concurrently with fiber/matrix damage, matrix deformation, and local load redistribution within the adopted RVE morphology.

For the RVE-fitted stiffnesses, the separations corresponding to damage initiation were 0.0926 μm under normal loading and 0.4566 μm under tangential loading. When the prescribed structural stiffness of 1.0 × 10^5^ MPa/mm is combined with the same effective strengths, the corresponding separations are 0.2743 and 0.3902 μm, respectively. Thus, the prescribed structural stiffness changes the pre-damage compliance and the separation at damage initiation relative to the fitted RVE response.

Three fiber arrangements and three mesh densities were analyzed, and the cohesive-zone length and the number of elements spanning the fracture process zone were not established. The properties reported in Table 9 are therefore specific to the adopted 40 × 40 × 40 μm^3^ morphology, mesh, and interfacial properties. The observed damage sequence characterizes the behavior of the adopted numerical realizations.

### 3.3. Opening-Dominated Asymmetric DCB Response and Model–Experiment Comparison

**Figure 11:**
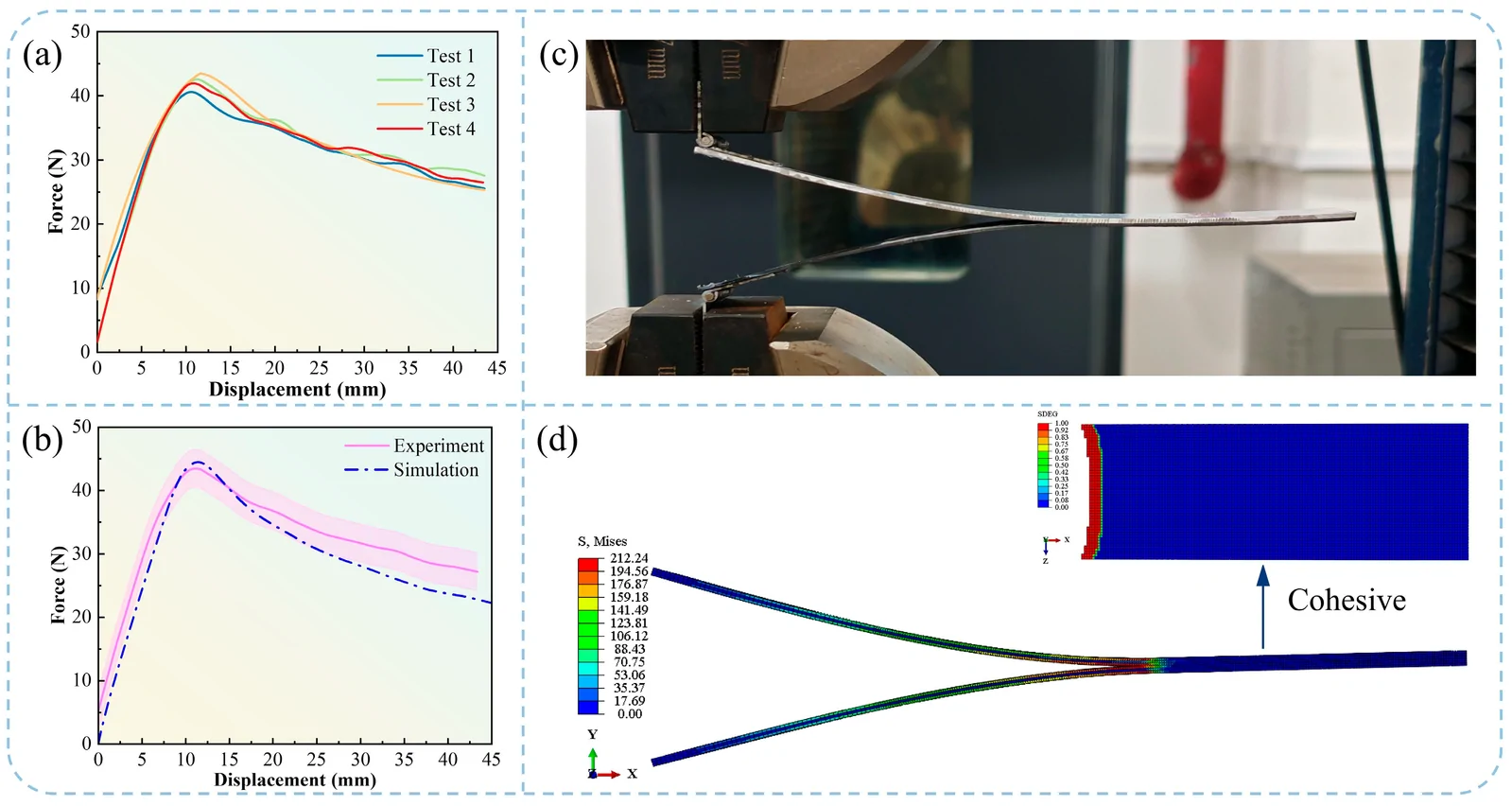
Opening-dominated asymmetric DCB response and model–experiment comparison for CARALL: (**a**) individual experimental load–displacement curves (n = 4); (**b**) pointwise experimental mean ± SD and deterministic FE response; (**c**) representative specimen deformation during opening; and (**d**) predicted cohesive damage along the Al/matrix interface.

Four opening-dominated asymmetric DCB specimens (n = 4), each containing the same nominal 50-mm PTFE starter crack, were used for the structural comparison. Their peak loads were 40.61, 42.56, 43.53, and 41.95 N, respectively, giving a mean of 42.16 N, a sample standard deviation of 1.22 N, and a coefficient of variation of 2.90%. The range was 40.61–43.53 N, and the two-sided 95% confidence interval was 40.22–44.11 N. Pointwise means and standard deviations were calculated at the common displacement coordinates of the four curves (Figure 11a,b). The close clustering of the four peak loads indicates consistent specimen fabrication and loading.

The deterministic FE analysis used the RVE-derived cohesive strengths and fracture energies without calibration against the DCB data, with the structural penalty stiffnesses prescribed independently. The calculated response followed the overall shape and peak-load region of the measured responses (Figure 11b), while the post-peak branch was smoother and lacked the local fluctuations associated with experimental crack propagation. The FE peak load was 44.48 N, 5.50% above the experimental mean of 42.16 N. The 5.50% difference exceeds the scatter of the four specimens (a 95% confidence interval of 40.22–44.11 N) and is comparable to the change produced by the prescribed ±20% perturbations of the transferred parameters below in this section; it therefore indicates that the predicted response lies in the same load range as the measurements and is not evidence that the transferred parameters reproduce the experimental response.

Mesh convergence was assessed by varying the bonded-region element size from 0.5 to 1.0 mm (Table 10). The predicted peak load remained between 44.18 and 45.47 N, with a mean of 44.76 ± 0.48 N (coefficient of variation 1.08%), indicating that the adopted 0.5 mm mesh lies within the converged range. The kinetic-to-internal-energy ratio remained below 1.5% for every mesh size, confirming that the explicit solution approached quasi-static conditions.

The cohesive-zone length was estimated as *l_cz_* ≈ *E*′·*G_Ic_*/*σ_c_*^2^. With *E*′ = 70 GPa, *G_Ic_* = 0.36 N/mm, and *σ_c_* = 27.42 MPa, this gives *l_cz_* ≈ 34 mm, so that the 0.5 mm bonded-region mesh spans approximately 68 elements across the process zone, resolving it adequately.

A one-at-a-time sensitivity analysis was also performed on the structural penalty stiffness and the transferred cohesive parameters (Table 11). Each parameter family was perturbed independently while all remaining parameters were held at their transferred values. Within a given perturbation, the normal and tangential strengths were scaled simultaneously by the same factor, and the Mode I and Mode II fracture energies were scaled simultaneously by the same factor; the normal and tangential components were therefore perturbed jointly rather than separately, and no joint or correlated perturbation of strength and fracture energy was examined. The peak load changed by 1.2% when the penalty stiffness was varied by factors of 0.5 and 2, by −1.5% and +0.9% when the cohesive strength was varied by factors of 0.8 and 1.2, and by −5.3% and +9.8% when the fracture energy was varied by the same factors. The predicted peak load is therefore insensitive to the penalty stiffness and the cohesive strength, but it responds more strongly to the fracture energy. Because the ±20% perturbations were prescribed rather than sampled from a distribution, and because each parameter was varied in isolation, these results characterize the sensitivity of the structural response over the investigated parameter range only; they do not constitute an uncertainty range, a propagated uncertainty budget, or a quantified confidence interval for the transferred parameter set.

Because the crack length was tracked qualitatively and not recorded at synchronized load–displacement points, the retained data do not support compliance calibration or ASTM D5528 initiation and propagation GI values and R-curves. The structural comparison was therefore restricted to the measured load–displacement responses, the peak-load distribution, and the qualitative damage location.

Macroscopic post-test inspection indicated that cracks propagated predominantly within the Al/matrix interfacial region, with epoxy residue remaining on parts of the aluminum surface. In the FE model, cohesive damage developed ahead of the starter crack and advanced along the bonded interface (Figure 11d). The predicted damage location and propagation direction were qualitatively consistent with the observations.

The aluminum and composite arms had different elastic and bending properties, so their unequal flexural rigidities could alter the crack-tip mode partition and the large opening displacement could introduce rotation-dependent effects. The experiment was therefore treated as an opening-dominated asymmetric DCB test.

The comparison of the global response and of the damage location supports the transfer procedure descriptively for the reported specimen and numerical configuration; it does not confirm the transferred values, because the predicted and measured load levels differ by more than the experimental scatter and the comparison is restricted to the load–displacement response and a qualitative damage location. The assessment is configuration-specific: the atomistic input was a single realization, the RVE input was evaluated for three stochastic fiber realizations of the same morphology, the Mode II descriptors were not examined with a structural Mode II or mixed-mode test, and no synchronized crack-length or independent fracture-resistance data were available for the experimental interface. These results therefore provide a configuration-specific assessment of the transfer procedure rather than a general validation of the multiscale workflow or of the transferred parameters. These results characterize structural sensitivity only. The interaction-strength study was performed at the atomistic level and was not propagated through the mesoscale RVE to the DCB model, so the present perturbations do not constitute a quantified uncertainty statement for the predicted peak load.

## 4. Conclusions

This study examined how far cohesive-zone parameters for an opening-dominated interlaminar fracture in CARALL can be transferred bottom-up, from an atomistic Al(111)/epoxy interface through a mesoscale RVE to a structural DCB model, without calibration against the structural test response.

At the atomistic scale, the idealized nonbonded Al(111)/epoxy interface exhibited normal and tangential peak tractions of 470.09 and 352.93 MPa at 0.005 Å/fs, with the peak tractions varying by less than 6% across 0.001–0.005 Å/fs. The corresponding Mode I and Mode II works of separation were 0.178 and 0.737 N/mm, so that the tangential response dissipated approximately four times more energy than the normal response while retaining a lower peak traction.

At the mesoscale, three independent stochastic fiber realizations of the same morphology produced closely consistent effective parameters, with mean normal and tangential strengths of 27.42 ± 0.33 and 39.04 ± 0.65 MPa and mean Mode I and Mode II fracture energies of 0.36 ± 0.02 and 0.81 ± 0.04 N/mm (coefficients of variation below 5%), and fiber/matrix damage preceded Al/matrix damage in every realization. Conversion through the heterogeneous RVE therefore attenuated the intrinsic Al/epoxy strength by approximately 94% and 89%, and this attenuation, rather than the atomistic strength itself, controls the effective interlaminar properties of the adopted morphology.

At the structural scale, the mesoscale parameters were assigned directly to the DCB cohesive layer without specimen-level calibration and, with independently prescribed penalty stiffnesses, predicted a peak load of 44.48 N, 5.50% above the four-specimen mean of 42.16 ± 1.22 N, together with an interfacial damage path that was qualitatively consistent with the observed failure location. The predicted peak load was insensitive to the penalty stiffness and to the cohesive strength but most sensitive to the fracture energy, which identifies the Mode I fracture energy of the interlaminar region as the parameter that warrants the most direct experimental characterization in follow-up work.

Because crack length was not recorded at synchronized load–displacement points and no independent fracture-resistance data were available, and because the atomistic interface idealizes the anodized surface, the present results are a configuration-specific assessment rather than a validated transfer route. Extending it to a predictive tool will require synchronized crack-length measurements, independent fracture-resistance data, and an atomistic representation that captures the oxide chemistry and morphology of the anodized interface.

## Figures and Tables

**Table 1:** Material properties of the constituents used in the mesoscale RVE [69,70].

Constituent	Density, ρ (g/cm^3^)	Elastic Modulus E (GPa)	Poisson’s Ratio, ν	Shear Modulus G (GPa)
5052 Al	2.68	70.3	0.33	Not applicable
Matrix	1.20	3.17	0.355	1.02
T700	1.80	E_11_ = 230	*ν*_12_ = 0.2	G_12_ = 24
		E_22_ = 15	*ν*_13_ = 0.2	G_13_ = 24
		E_33_ = 15	*ν*_23_ = 0.25	G_23_ = 18.2

**Table 2:** Prescribed fiber/matrix cohesive parameters used in the mesoscale RVE [69].

Interface and Component	Stiffness (MPa/μm)	Initiation Strength (MPa)	Fracture Energy (μN/μm)
Fiber/matrix	*K_nn_* = 1.0 × 10^5^	*t_n_*_,0_ = 45	*G_Ic_* = 0.02
	*K_ss_* = *K_tt_* = 1.0 × 10^5^	*t_s_*_,0_ = *t_t_*_,0_ = 45	*G_IIc_* = *G_IIIc_* = 0.15

**Table 3:** CFRP material parameters used in the macroscale FE model [72].

Parameter Type	Value
Density	ρ = 1780 kg/m^3^
Elastic modulus	E_11_ = 114,000 MPa; E_22_ = E_33_ = 8610 MPa
Shear modulus	G_12_ = G_13_ = 4160 MPa; G_23_ = 3000 MPa
Poisson’s ratio	ν_12_ = ν_13_ = ν_23_ = 0.3
Strength parameter	*X_t_* = 2688 MPa; *X_c_* = 1458 MPa; *Y_t_* = 69.5 MPa; *Y_c_* = 236 MPa; *Z_t_* = 55.5 MPa; *Z_c_* = 175 MPa; *S*_12_ = 136 MPa; *S*_23_ = 95.5 MPa

**Table 4:** Effect of the imposed MD loading rate on the peak tractions and traction–separation integrals of the idealized Al/epoxy interface.

Rate (Å/fs)	Normal Peak Traction (MPa)	Mode I Integral (MPa Å)	Shear Peak Traction (MPa)	Mode II Integral (MPa Å)
0.001	443.76	1607.07	333.52	7916.48
0.002	450.11	1807.80	339.16	7502.34
0.003	456.93	1745.11	344.46	8613.59
0.004	463.74	2113.23	349.04	6948.78
0.005	470.09	1778.71	352.93	7373.55

**Table 5:** Sensitivity of the MD interface descriptors to the Al/epoxy Lennard-Jones interaction strength ε.

*ε* Scaling Factor	Normal Peak Traction *σ_n,peak_* (MPa)	Tangential Peak Traction *σ_t,peak_* (MPa)	Mode I Work of Separation (MPa·Å)	Mode II Work of Separation (MPa·Å)
0.5	187.98	253.79	495.96	3390.04
1.0	470.09	352.93	1778.71	7373.55
1.5	730.92	442.23	4414.95	12,894.37

**Table 6:** Model-specific Al/epoxy cohesive descriptors extracted from MD simulations and converted to the RVE unit system.

Parameter Type	Symbol	MD Output	Converted RVE Input
Normal strength	*t_n_*_,0_	470.09 MPa	470.09 MPa
Tangential strength	*t_s_*_,0_ = *t_t_*_,0_	352.93 MPa	352.93 MPa
Mode I fracture energy	*G_Ic_*	1778.71 MPa Å	0.1778 μN/μm
Mode II fracture energy	*G_IIc_*	7373.55 MPa Å	0.7373 μN/μm
Normal stiffness	*K_nn_*	940.16 MPa/Å	9.4016 × 10^6^ MPa/μm
Tangential stiffness	*K_ss_* = *K_tt_*	235.29 MPa/Å	2.3529 × 10^6^ MPa/μm

**Table 7:** Effective cohesive parameters of the three stochastic fiber realizations of the mesoscale RVE, together with their means and standard deviations.

Realization	*σ_n_* (MPa)	*σ_t_* (MPa)	*G_Ic_* (N/mm)	*G_IIc_* (N/mm)
1	27.75	39.70	0.36	0.84
2	27.43	39.02	0.37	0.82
3	27.09	38.40	0.34	0.77
Mean ± SD	27.42 ± 0.33	39.04 ± 0.65	0.36 ± 0.02	0.81 ± 0.04

**Table 8:** Mesh-convergence study of the mesoscale RVE, performed for a single realization.

Element Size (μm)	*σ_n_* (MPa)	*σ_t_* (MPa)	*G_Ic_* (N/mm)	*G_IIc_* (N/mm)
0.5	27.45	40.00	0.41	0.85
0.8	27.43	39.02	0.37	0.82
1.2	27.40	38.25	0.42	0.83

**Table 9:** Effective cohesive parameters and prescribed penalty stiffnesses transferred to the macroscale finite-element model.

Effective Parameter	Symbol	Value	Unit
Normal stiffness	*K_nn_*	1.0 × 10^5^	MPa/mm
Tangential stiffness	*K_ss_ = K_tt_*	1.0 × 10^5^	MPa/mm
Normal strength	*t_n_*_,0_	27.42	MPa
Tangential strength	*t_s_*_,0_ *= t_t_*_,0_	39.04	MPa
Mode I fracture energy	*G_Ic_*	0.36	N/mm
Mode II fracture energy	*G_IIc_*	0.81	N/mm

**Table 10:** Mesh-convergence study of the asymmetric DCB model.

Bonded-Region Element Size (mm)	Peak Load (N)	KE/IE Ratio (%)
0.5	44.48	0.31
0.6	44.18	0.43
0.7	45.18	0.79
0.8	44.73	0.99
0.9	44.50	1.07
1.0	45.47	1.41

**Table 11:** One-at-a-time sensitivity of the predicted peak load to the structural penalty stiffness and the transferred cohesive parameters of the asymmetric DCB model.

Parameter	Variation	Peak Load (N)
Penalty stiffness K	×0.5	44.44
	×2	44.97
Cohesive strength *σ_c_*	×0.8	43.83
	×1.2	44.46
Fracture energy G	×0.8	42.12
	×1.2	48.85

## Data Availability

Data are contained within the article. Further inquiries can be directed to the corresponding author.
